# Possible Participation of Ionotropic Glutamate Receptors and l-Arginine-Nitric Oxide-Cyclic Guanosine Monophosphate-ATP-Sensitive K^+^ Channel Pathway in the Antinociceptive Activity of Cardamonin in Acute Pain Animal Models

**DOI:** 10.3390/molecules25225385

**Published:** 2020-11-18

**Authors:** Chung Pui Ping, Muhammad Nadeem Akhtar, Daud Ahmad Israf, Enoch Kumar Perimal, Mohd Roslan Sulaiman

**Affiliations:** 1 Department of Biomedical Sciences, Faculty of Medicine and Health Sciences, University Putra Malaysia, Serdang 43400, Selangor, Malaysia; puiping.chung@gmail.com (C.P.P.); daudaia@upm.edu.my (D.A.I.); enoch.perimal@adelaide.edu.au (E.K.P.); 2 Faculty of Industrial Sciences & Technology, University Malaysia Pahang, Gambang 26300, Pahang, Malaysia; nadeemupm@gmail.com; 3 Laboratory of Natural Products, Institute of Bioscience, University Putra Malaysia, Serdang 43400, Selangor, Malaysia; 4 Australian Research Council Centre of Excellence for Nanoscale BioPhotonics, University of Adelaide, Adelaide 5000, Australia

**Keywords:** cardamonin, glutamate receptor, l-arginine nitric oxide pathway, cGMP, potassium channels, acute pain

## Abstract

The perception of pain caused by inflammation serves as a warning sign to avoid further injury. The generation and transmission of pain impulses involves various pathways and receptors. Cardamonin isolated from *Boesenbergia rotunda* (L.) Mansf. has been reported to exert antinociceptive effects in thermal and mechanical pain models; however, the precise mechanism has yet to be examined. The present study investigated the possible mechanisms involved in the antinociceptive activity of cardamonin on protein kinase C, *N*-methyl-d-aspartate (NMDA) and non-NMDA glutamate receptors, l-arginine/cyclic guanosine monophosphate (cGMP) mechanism, as well as the ATP-sensitive potassium (K^+^) channel. Cardamonin was administered to the animals intra-peritoneally. Present findings showed that cardamonin significantly inhibited pain elicited by intraplantar injection of phorbol 12-myristate 13-acetate (PMA, a protein kinase C activator) with calculated mean ED_50_ of 2.0 mg/kg (0.9–4.5 mg/kg). The study presented that pre-treatment with MK-801 (NMDA receptor antagonist) and NBQX (non-NMDA receptor antagonist) significantly modulates the antinociceptive activity of cardamonin at 3 mg/kg when tested with glutamate-induced paw licking test. Pre-treatment with l-arginine (a nitric oxide precursor), ODQ (selective inhibitor of soluble guanylyl cyclase) and glibenclamide (ATP-sensitive K^+^ channel inhibitor) significantly enhanced the antinociception produced by cardamonin. In conclusion, the present findings showed that the antinociceptive activity of cardamonin might involve the modulation of PKC activity, NMDA and non-NMDA glutamate receptors, l-arginine/nitric oxide/cGMP pathway and ATP-sensitive K^+^ channel.

## 1. Introduction

Cardamonin, a natural occurring chalcone, which is chemically known as 2′,4′-dihydroxy-6′-methoxychalcone (C_16_H_14_O_4_), was initially isolated from the seeds of *Amomum subulatum* [1]. To date, cardamonin isolated from *Alpinia rafflesiana*, *Alpinia henryi*, *Alpinia katsumadai*, and *Campomanesia adamantium* has been extensively studied. Cardamonin was reported to exert anti-inflammatory properties [2,3], vasorelaxation of rat mesenteric artery [4], anti-proliferative activity and induced apoptosis [5,6,7], protection against acute lung injury in septic mice [8], anti-mutagenic effects [9], improved insulin resistance in high fructose-fed rat model [10], pigmentation inhibitory effect in normal human melanocytes [11], inhibition against pruritis [12], nephroprotective effect against cisplatin-induced renal injury [13], and inhibition of the differentiation of preadipocytes into adipocytes [14]. Cardamonin showed promising effects in a number of cancer studies, involving colon, breast, ovarian and gastric cancer cell lines [15,16,17,18,19,20,21,22]. In vivo study of cardamonin treatment against rheumatoid arthritis presented inhibition on inflammation as well as pain through behavioural, biochemical and histological studies; plasma evaluation of the treated animals also showed promising inhibition in pro-inflammatory cytokine levels [23]. Therapeutic effect of cardamonin on chronic constriction injury-induced neuropathic pain has been reported through a series of thermal and mechanical pain studies [24].

Both in vitro and ex vivo studies reported that cardamonin possessed inhibitory action against pro-inflammatory cytokine production [2,25]. Further studies reported the inhibitory action of cardamonin against inflammatory responses involved in the disruption of nitric oxide (NO) production and the downregulation of the iNOS expression via modulation of the NF-ҡB pathway [3,25,26]. An in vivo study with lipopolysaccharide (LPS)-challenged ICR mice model reported that cardamonin also suppressed the generation of nitric oxide [27]. A previous study presented that cardamonin showed antinociceptive activity against PBQ-induced writhing and carrageenan-indyced hyperalgesia [28,29].

Taking all these into account, a deeper understanding of the mechanism of antinociceptive activity of cardamonin has to be carried out. In a previous study, cardamonin demonstrated antinociceptive activity through a acetic acid-induced abdominal writing test, hot plate test and glutamate-induced nociception tests [29]. Activation of *N*-methyl-d-aspartate (NMDA) and non-NMDA glutamate receptors is probably involved in the glutamate-induced nociception. In particular, activation of NMDA receptor is mediated by the l-arginine-nitric oxide-cyclic GMP pathway [30,31]. Thus, in the present study, we attempted to study the possible participation of ionotropic glultamate receptors and nitric oxide/cyclic GM/ATP-sensitive K^+^ channel pathway in the antinociceptive activity of cardamonin.

## 2. Results

### 2.1. Antinociceptive Analysis

#### 2.1.1. Acetic Acid-Induced Abdominal Writhing Test

**Figure 1:**
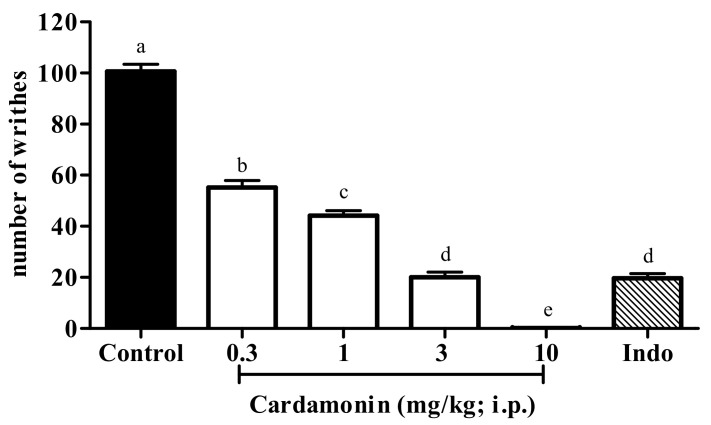
Effect of cardamonin (0.3, 1, 3 and 10 mg/kg; i.p.) against acetic acid-induced nociception. Each column represents the mean ± S.E.M. of six mice. Control group received only the vehicle (ethanol: Tween 20: distilled water in 5:5:90, *v*/*v*) used to dilute the compound. Indomethacin (Indo; 10 mg/kg; i.p.) was used as positive control. Statistical analysis was determined by one-way ANOVA, followed by Tukey’s post hoc test. Values with different superscript letters are statistically different from each other at *p* < 0.05.

The result in Figure 1 presented the effect of systemically administered cardamonin in acetic acid-induced abdominal writhing test. Cardamonin at the dose of 0.3, 1, 3 and 10 mg/kg produced significant dose-dependent inhibition against acetic acid-induced pain, with the percentage of inhibition at 45%, 56%, 80% and 100%, respectively. For the use throughout the experiments in the mechanism studies, the calculated mean ED_50_ value for intraperitoneal administration of cardamonin was 2.1 mg/kg (1.9–2.5 mg/kg). Indomethacin (Indo; 10 mg/kg; i.p.), which served as the positive control drug, showed significant inhibition, with 80% of inhibition against acetic acid-induced pain in mice, according to our previously published results [29].

#### 2.1.2. Involvement of Protein Kinase C

**Figure 2:**
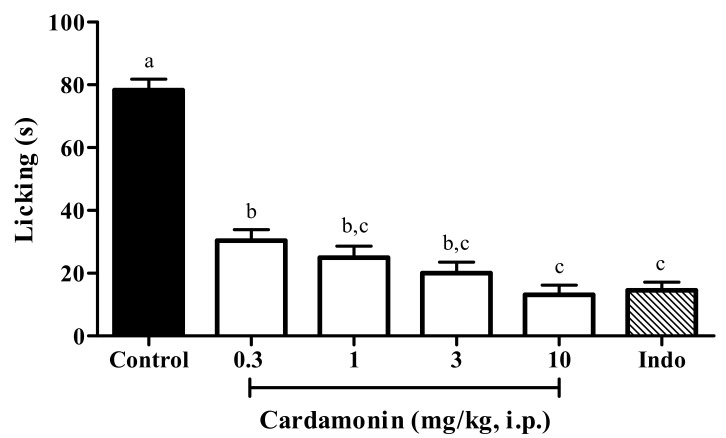
Effect of cardamonin (0.3, 1, 3 and 10 mg/kg; i.p.) against phorbol 12-myristate 13-acetate (PMA)-induced nociception. Each column represents the mean ± S.E.M. of 6 mice. Control group receives only the vehicle used to dilute the compound. Indomethacin (Indo; 10 mg/kg; i.p.) was used as the positive control drug. Statistical analysis was determined by one-way ANOVA, followed by Tukey’s post hoc test. Values with different superscript letters are statistically different from each other at *p* < 0.05.

The intraperitoneal administration of cardamonin at doses of 0.3, 1, 3 and 10 mg/kg demonstrated significant dose dependent inhibition in phorbol 12-myristate 13-acetate (PMA)-induced paw licking test in mice, with 61%, 68%, 74% and 83% of inhibition respectively (Figure 2). The calculated mean ED_50_ value for this study was 2.0 mg/kg (CI, 0.9–4.5 mg/kg). Indomethacin (Indo; 10 mg/kg; i.p.) was used as the positive control drug and it showed significant inhibition as compared to the control group, with 81% of inhibition against PMA-induced nociception.

#### 2.1.3. Effect of MK-801 and NBQX on Glutamate-Induced Nociception

**Figure 3:**
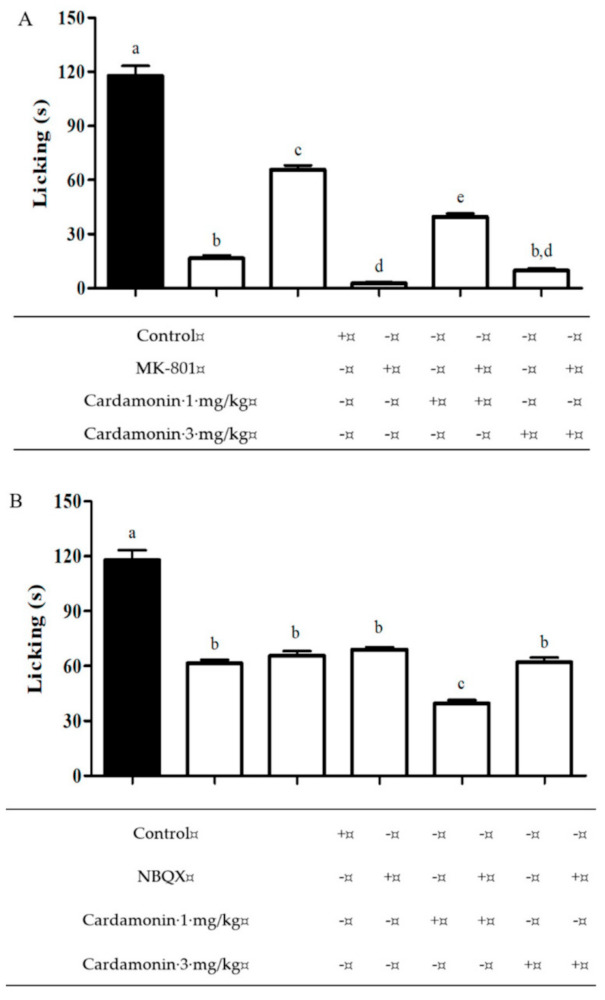
Effect of MK-801 (0.3 mg/kg; i.p.) (panel **A**) and NBQX (3 mg/kg; i.p.) (panel **B**) on antinociception caused by cardamonin (1 and 3 mg/kg; i.p.) in glutamate-induced paw licking test. Each column represents the mean ± S.E.M. of 6 mice. Statistical analysis was determined by one-way ANOVA, followed by Tukey’s post hoc test. Values with different superscript letters are statistically different from each other at *p* < 0.05.

Figure 3 presented the antinociceptive effect of cardamonin on NMDA glutamate receptor subtype (Panel A) and non-NMDA glutamate receptor subtype (Panel B) when assessed in glutamate-induced paw licking test. NMDA receptor antagonist, MK-801 (0.3 mg/kg; i.p.) produced significant inhibition when administered alone intraperitoneally, with 86% of inhibition in paw licking when compared to the control group. Pre-treatment with MK-801 prior to the administration of cardamonin (1 and 3 mg/kg) significantly enhanced the effect of the cardamonin treatment alone respectively. Administration of AMPA/kainate receptor antagonist, NBQX (3 mg/kg; i.p.) alone produced significant inhibition in glutamate-induced nociception, with 48% of inhibition when compared to the control group. Pre-treatment with NBQX produced no significant changes for the treatment with cardamonin 1 mg/kg when compared to the treatment alone, but when treated with cardamonin 3 mg/kg, NBQX significantly reversed the antinociceptive effect of cardamonin.

**Figure 4:**
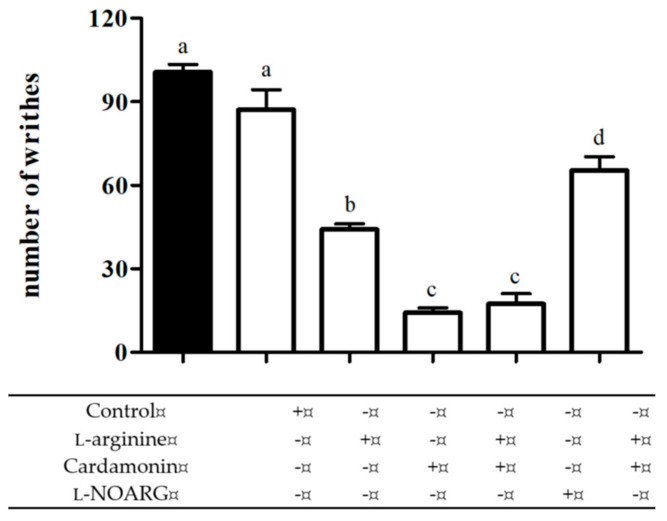
Effect of pre-treatment with l-arginine (100 mg/kg; i.p.) on antinociceptive activity of cardamonin (1 mg/kg; i.p.) and l-NOARG (20 mg/kg; i.p.) against acetic acid-induced abdominal writhing test in mice. Each column represents the mean ± S.E.M. of six mice. Statistical analysis was determined by one-way ANOVA, followed by Tukey’s post hoc test. Values with different superscript letters are statistically different from each other at *p* < 0.05.

**Figure 5:**
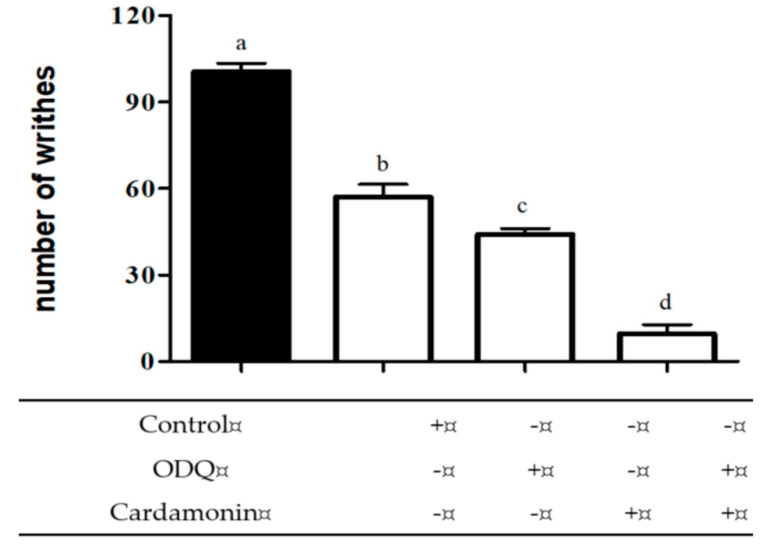
Effect of ODQ (2 mg/kg; i.p.) pre-treatment on antinociceptive activity of cardamonin (1 mg/kg; i.p.) against acetic acid-induced abdominal writhing test in mice. Each column represents the mean ± S.E.M. of six mice. Statistical analysis was determined by one-way ANOVA, followed by Tukey’s post hoc test. Values with different superscript letters are statistically different from each other at *p* < 0.05.

**Figure 6:**
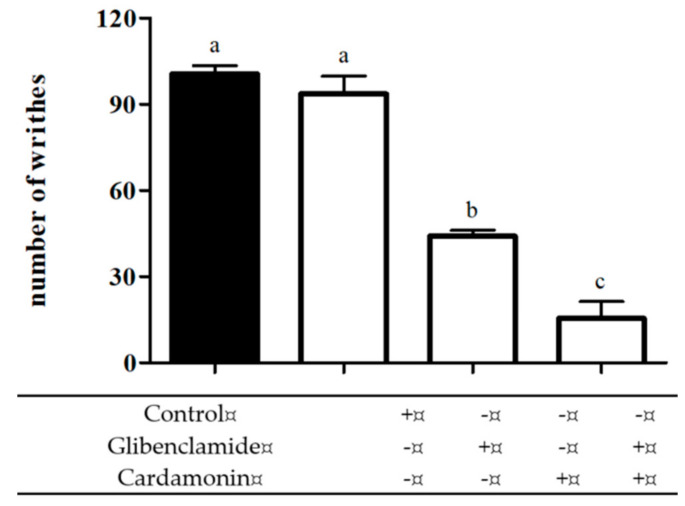
Effect of glibenclamide (10 mg/kg; i.p.) pre-treatment on antinociceptive activity of cardamonin (1 mg/kg; i.p.) against acetic acid-induced pain in mice. Each column represents the mean ± S.E.M. of six mice. Statistical analysis was determined by one-way ANOVA, followed by Tukey’s post hoc test. Values with different superscript letters are statistically different from each other at *p* < 0.05.

All Figure 3, Figure 4, Figure 5 and Figure 6 are misaligned as indicated by yellow highlights. Please check attached original manuscript file.

### 2.2. Analysis of the Possible Mechanism of Action of Cardamonin

#### 2.2.1. Involvement of l-arginine/Nitric Oxide Pathway

The result depicted in Figure 4 showed that pre-treatment with nitric oxide precursor, l-arginine (100 mg/kg; i.p.), at a dose that produced no significant different as compared to the control group, significantly reversed the antinociception exhibited by the nitric oxide synthase inhibitor, l-NOARG (20 mg/kg; i.p.) and significantly enhanced the antinociceptive effect of cardamonin (1 mg/kg; i.p.), when analysed with an acetic acid-induced nociceptive test.

#### 2.2.2. Involvement of Cyclic Guanosine Monophosphate (cGMP)

The result depicted in Figure 5 showed the effect of cardamonin upon the injection of the specific guanylyl cyclise inhibitor, ODQ (2 mg/kg; i.p.) and analysed with acetic acid-induced nociceptive test. Both ODQ and cardamonin, when administered alone, produced significant inhibition in acetic acid-induced abdominal writhing test. However, when given together, ODQ significantly enhanced the antinociceptive effect of cardamonin compared to the treatment alone.

#### 2.2.3. Involvement of ATP-Sensitive K^+^ Channel

The pre-treatment with ATP-sensitive K^+^ channel inhibitor, glibenclamide (10 mg/kg; i.p.), produced no significant difference as compared with the control group when administered alone, but significantly enhanced the antinociceptive effect of cardamonin (1 mg/kg; i.p.) in the acetic acid-induced abdominal writhing test (Figure 6).

## 3. Discussion

The perception of pain involves various pathways which transmit the pain impulses from the site of injury to the peripheral nervous system then central nervous system. A previous study showed that cardamonin exhibited antinociceptive action by interrupting the opioidergic pathway and TRPA1 activation [24,32]. Cardamonin was reported to inhibit nociception through action on TRPV1 channel and glutamate receptors [29]. The stimulation of nerve endings involves the release of various inflammatory mediators which lead to sensitization of respective receptors embedded on the neurons surface [33].

Prostaglandin E_2_ sensitization of TRPV1 activity in mice involves protein kinase C (PKC)-dependent pathway [34]. The findings of the present study shows that the antinociceptive effect of cardamonin involved inhibition of protein kinase C activity. The systemic administration of cardamonin exhibited significant dose dependent inhibition against overt nociception induced by phorbol-12-myristate-13-acetate (PMA)-induced paw licking test. The protein kinase C signalling pathway plays an important role in regulating the excitation of sensory neurons through the phosphorylation of membrane-bound receptors and ion channels. The injection of PMA, a phorbol ester which represent pharmacological analogues of diacylglycerol, into the mouse paw through intraplantar injection, directly activates protein kinase C [35]. The nociceptive behavioural responses induced by the injection of PMA into mouse paw attributed to translocation of protein kinase C isoforms from cytoplasmic region to peripheral ending of primary afferent nerves [35,36]. Thus, it was postulated that cardamonin might inhibit the translocation of protein kinase C to the primary afferent nerve endings, which then reduced peripheral nociception. Previous study reported that activation of protein kinase C potentiates the effect of capsaicin on TRPV1 receptor [37]. Protein kinase C mediated sensitization of TRPV1 receptor enhances glutamatergic synaptic transmission at the central terminal of sensory neurons in the dorsal horn of spinal cord [38]. At the central level, cardamonin might have the capability to interfere with the binding of protein kinase C to TRPV1 receptor and thus reduces the influx of calcium, as well as decreases glutamate activity, resulting in inhibition upon paw licking behavioural responses.

Cardamonin has been reported to exert inhibition of pain behaviour against glutamate-induced nociceptive test in mice [29]. There are three subtypes of ionotropic glutamate receptors, namely α-amino-3-hydroxy-5-methyl-4-isoxazolepropionic acid (AMPA), kainate and *N*-methyl-d-aspartate (NMDA) glutamate receptors. The AMPA receptors are thought to mediate rapid excitatory neurotransmission in the central nervous system in the earlier findings, but recent studies have demonstrated that spinal AMPA receptors contribute in the development of both acute and painful responses. The C-terminal intracellular regulatory domain of AMPA receptor subunit presents multiple phosphorylation sites for various protein kinases participate in the regulation of AMPA receptor function. The activation of protein kinase cascades, including calcium/calmodulin protein kinase II (CaMKII), protein kinase C (PKC) and protein kinase A (PKA) by noxious stimulation in the periphery tissues, may play a crucial role in the phosphorylation of glutamate receptors in spinal nociceptive neurons, which then followed by enhanced activity of glutamatergic synapses [39].

In the present study, animals were treated with quinoxalinedione antagonist, NBQX to block out glutamate binding to the non-NMDA receptors, but this antagonist has a limitation in distinguishing AMPA from kainate receptors [40]. The present findings suggest that cardamonin at the dose of 3 mg/kg exerted its antinociceptive property, possibly by blocking the binding of glutamate to AMPA receptor and inhibition of protein kinase cascades, in particular, protein kinase C-mediated phosphorylation of glutamate receptors, which then lead to reduced activity at glutamatergic synapses.

The activation of NMDA glutamate receptor in chronic pain settings happens at peripheral, spinal and supraspinal level of neural axis [41]. Peripheral administration of NMDA receptor antagonists attenuated the nociceptive behaviours caused by local injections of glutamate or NMDA, which shows there is the presence of NMDA receptors in the periphery [42]. The present study reported that systemic injection of NMDA receptor antagonist could reduce the nociceptive behaviours caused by local injection of glutamate into mouse paw; in which, intraperitoneal administration of MK-801 (NMDA receptor antagonist) showed significant inhibition in glutamate-induced paw licking behaviour when compared to the control group. Consistent with these findings, MK-801 also produced a dose dependent inhibition in glutamate-induced nociception when administered at periphery, systemic, spinal and supraspinally [30]. Despite the antinociceptive property, NMDA receptor antagonists also caused disturbance in motor coordination, but these two effects cannot be separated [30,43].

This study presented that cardamonin showed significant inhibition but not as effective as MK-801 when tested in glutamate-induced paw licking test. When pre-treated with MK-801, cardamonin was able to produce significant enhancement of inhibitory effect when compared to their respective dose treatment of cardamonin. These findings suggested that the inhibitory effect of cardamonin might involve the blocking NMDA receptor. Thus, the present study postulated that the antinociceptive effect of cardamonin in glutamate-induced nociception might involve the modulation of the activity of ionotropic glutamate receptor, including NMDA, AMPA and kainate receptors.

Nitric oxide is a diffusible gas that functions as a neurotransmitter in the pain processing pathway. The catalytic action performed by nitric oxide synthase (NOS), generates nitric oxide from l-arginine and molecular oxygen [44]. The administration of l-arginine systemically into the peritoneal region provided substrates for the action of the enzyme nitric oxide synthase (NOS) to produce a sufficient amount of nitric oxide to induce pain-related behaviour when examined with acetic acid-induced abdominal constriction model. Thus, in order to control the synthesis of nitric oxide, one must regulate the activity of its producing enzyme, which is the nitric oxide synthase (NOS) [45].

Previous studies reported that the administration of nitric oxide synthase inhibitors, both systemically and intrathecally, are capable to reduce hyperalgesia [46,47]. Furthermore, it was reported that l-NG-nitro-arginine (l-NOARG), which is a selective inhibitor of nitric oxide biosynthesis, showed antinociceptive activity when tested in a mouse model [48]. In the present study, systemic administration of N^ω^-nitro-l-arginine (l-NOARG) has reduced the pain induced by acetic acid, as shown by the reduced abdominal constriction behaviour of the tested animals. Upon administration of the selective inhibitor of nitric oxide synthase (NOS), l-NOARG inhibited the synthesis of nitric oxide by inactivating the enzyme. Thus, disturbance in the downstream signalling by nitric oxide that regulates various ion channels leads to the cessation of pain processing. On the other hand, when l-arginine was administered prior to the selective inhibitor of nitric oxide synthase, l-NOARG, the inhibitory effect of l-NOARG, was reversed as the nitric oxide produced sufficient enough to induce pronociceptive behaviour.

In order to determine whether the antinociceptive pathway of cardamonin involves the inhibition of nitric oxide signalling, the animals were pre-treated with l-arginine prior to the systemic administration of cardamonin. The results showed that the group cardamonin pre-treated with l-arginine exhibited significantly enhanced inhibition in acetic acid-induced pain behavioural study when compared with the group treated with cardamonin only. Since cardamonin was postulated to block the NMDA receptor, the influx of calcium ion into the intracellular cavity is reduced; thus, the calcium/calmodulin-dependent nitric oxide synthase (NOS) fails to catalyse l-arginine into nitric oxide and l-citrulline, which then leads to disruption of pain signalling. It was also suggested that downstream signalling of nitric oxide pain signalling pathway involves the release of glutamate [49]. Thus, the present study suggested that the enhanced antinociceptive effect of cardamonin when pre-treated with l-arginine probably involves the modulation of the glutamate receptors.

The administration of ODQ, a selective inhibitor of soluble guanylyl cyclase, was expected to reduce the synthesis of cGMP, and thus reduce the behavioural response against pain. In the group of animals treated with ODQ prior to cardamonin, it produced enhanced antinociceptive activity with a decrease in abdominal writhing response, as compared to the cardamonin only treated group. This study postulated that the enhanced inhibitory effect may be attributed to the synergistic effect of both ODQ and cardamonin. The soluble guanylyl cyclase inhibitor, ODQ should reduce the synthesis of cGMP; the downstream signalling pathway may lead to pain processing; with additional help from cardamonin that has shown its inhibitory effect against glutamate, nitric oxide and other neurotransmitters, the inhibitory action in the nociceptive behavioural study has improved as compared with their individual treatment respectively. Furthermore, it was reported that ODQ significantly decreased the glutamate concentration and reduced the intensity of NMDA-induced pain-related behaviour [49]. Thus, cardamonin was postulated to exert its antinociceptive activity by inhibiting the NMDA activation evoked by nitric oxide/cGMP/glutamate release cascade.

The activation of nociceptors initiates an increase in inward currents by activating the non-potassium channels and/or reduction in outward currents that leads to membrane depolarization, followed by the generation of action potential. The membrane depolarization and excitation of dorsal root ganglion neurons by nociceptive stimuli provide a basis for the manifestation of ATP-sensitive potassium channel opening. The opening of potassium channels plays a pivotal role in regulating resting membrane potential and action potential firing threshold [50,51].

Glibenclamide is one of the sulfonylurea drugs that bind to sulfonylurea receptor (SUR) protein and affect the opening of ATP-sensitive potassium channels [52]. Previous studies reported that sulfonylurea drugs, such as glibenclamide, cause neither hyperalgesia nor antinociceptive activity when tested individually [53]. Furthermore, administration of glibenclamide alone did not affect the pain behaviour that arises from formalin injection, at both phase 1 and phase 2 [54]. The results of this study were in agreement with these evidences, in which glibenclamide, when injected alone, did not caused any changes in pain behaviour when tested with acetic acid-induced abdominal writhing test as compared to the control group. Pre-treatment of glibenclamide has blocked the opening of ATP-sensitive potassium channels, but when treated with cardamonin, the pain behaviour induced by acetic acid has greatly reduced and was significantly different from the group pre-treated with vehicle.

Nitric oxide plays dual effects on nociception and antinociception. The antinociceptive effects of nitric oxide involves the activation of ATP-sensitive potassium channels [55]. Nitric oxide binding to the soluble guanlyl cyclase (sGC) catalyzes guanosine triphosphate (GTP) forming 3′,5′-cyclic monophosphate (cGMP), followed by the activation of cGMP-dependent protein kinase (PKG). The ATP-sensitive potassium channels phosphorylated by PKG leads to membrane potential hyperpolarization, thus resulting in cessation of nociceptive signal transmission [56,57]. In addition, a study showed that nitric oxide was capable of up-regulating the expression of ATP-sensitive potassium channel in primary sensory neurons [58]. The findings in the present study suggested that cardamonin might facilitate in the up-regulation of ATP-sensitive potassium channels expression in the presence of nitric oxide.

## 4. Methodology

### 4.1. Plant Material

The fresh rhizomes of *Boesenbergia rotunda* (5 kg) were commercially purchased from the local market, Serdang, Malaysia, and were authenticated by a resident botanist at the Institute of Bioscience (IBS), Universiti Putra Malaysia (UPM). A voucher specimen (SK1780/10) was deposited at the Herbarium, which was located at Laboratory of Natural Products, IBS, UPM. A small part of the rhizomes was cultivated in the Medicinal Plant Garden at IBS, UPM for future reference.

### 4.2. Extraction and Isolation

The fresh rhizomes of *Boesenbergia rotunda* were sliced into small flat pieces and dried under shadow for one week. The dried rhizomes were ground into fine powder by using domestic food processor. The dried powder 2.5 kg was dissolved in distilled methanol for two to three days. The methanolic extract was filtered and concentrated on rotary evaporator and 255 g of crude extract was obtained. The methanolic extract was subjected to solvent extraction. The crude methanolic extract was dissolved in 250 mL distilled water and transferred into a separating funnel. About 150 mL hexane was added into aqueous layer and subsequently extracted with chloroform, ethyl acetate and butanol. The chloroform layer finally passed over sodium sulphate anhydrous to remove the moisture. The chloroform extract was subjected to flash column chromatography by using ethyl acetate and hexane as eluents. Finally, the compound was purified from chloroform extract and identified as cardamonin after performing the detailed spectroscopic techniques [23,29].

### 4.3. Experimental Animals

Adult male ICR mice (20–30 g) were used throughout the study. Animals were randomly divided into six mice per group (*n* = 6) and were housed at the Animal House of Faculty of Medicine and Health Sciences, Universiti Putra Malaysia. The condition of housing was set at 12 h light/12 h dark cycle with free access to standard pellet and water ad libitum. Animals were acclimatized to the condition of the laboratory one hour before the experiments. All of the animals were used only once in the experiment. Handling of animals and experiments was conducted according to the Ethical Guidelines for Investigation of Experimental Pain in Conscious Animals (Zimmermann, 1983) by the International Association for the Study of Pain (IASP). This study has been approved by the Institutional Animal Care and Use Committee (IACUC) UPM (Ref: UPM/FPSK/PADS/BR-UUH/00425).

### 4.4. Drugs and Chemicals

The following reagents and drugs were used: Tween 20, absolute ethanol, DMSO, indomethacin, MK-801, NBQX, glutamate, l-arginine, N^ω^-nitro-l-arginine (l-NOARG), 1H-[1,2,4]Oxadiazolo[4,3-a]quinoxalin-1-one (ODQ), phorbol 12-myristate 13-acetate (PMA), and glibenclamide. All the chemicals and drugs mentioned were purchased from Sigma Chemical Co. (USA). All drugs were dissolved in physiological saline (0.9% NaCl) unless otherwise mentioned. ODQ was dissolved in 5% DMSO and PMA was dissolved in phosphate buffered saline (PBS). Cardamonin and indomethacin were dissolved in ethanol, Tween 20 and distilled water in 5:5:90 (*v*/*v*) fractions. Respective controls received only solvent vehicle, whereby it had no effect per se on nociceptive responses. All drugs and cardamonin solution were freshly prepared and administered intraperitoneally (i.p.) in a volume of 10 μ/kg, unless otherwise stated in the method.

### 4.5. Antinociceptive Analysis

#### 4.5.1. Acetic Acid-Induced Abdominal Writhing Test

The acetic acid-induced abdominal writhing test was carried out as previously described [59], with slight modifications. Animals were pre-treated with cardamonin (0.3, 1, 3 and 10 mg/kg; i.p.), 30 min before the challenge with injection of 0.8% acetic acid (10 mL/kg; i.p.). Doses of cardamonin used was reported does not cause any apparent qualitative toxicity and disruption in motor coordination on animals [29]. The control group received a similar volume of vehicle (10 mL/kg; i.p.). Indomethacin (Indo, 10 mg/kg; i.p.) was used as the reference drug. Following the injection of acetic acid, the animals were immediately placed into a Perspex chamber and the number of abdominal writhes was recorded for 30 min, beginning 5 min after the acetic acid injection.

#### 4.5.2. Involvement of Protein Kinase C

The experiment was carried out as previously described [60,61]. A volume of 20 µL of phorbol 12-myristate 13-acetate (PMA; 0.03 µg/paw) was injected intraplantarly (i.p.) into the ventral surface of the right hind paw. Animals were individually placed into an observation chamber and were observed from 15 to 45 min following PMA injection. The amount of time spent licking and biting the injected paw was timed with a chronometer and was considered to be indicative of pain. The animals were treated with cardamonin (0.3, 1, 3 and 10 mg/kg; i.p.), indomethacin (10 mg/kg; i.p.) or vehicle (10 mg/kg; i.p.) 30 min before PMA injection.

#### 4.5.3. Effect of MK-801 and NBQX on Glutamate-Induced Nociception

To investigate the possible participation of NMDA receptor in the antinociceptive effect of cardamonin, the procedure used was similar to that previously described [30,62,63], with slight modifications. The animals were pre-treated with MK-801 (0.3 mg/kg; i.p.; NMDA receptor antagonist) or NBQX (3 mg/kg; i.p.; non-NMDA receptor antagonist) 15 min before injection of either vehicle (10 mL/kg; i.p.) or cardamonin (1 and 3 mg/kg; i.p.). The animals were then challenged with the injection of 20 µL of glutamate (10 µmol/paw) into the ventral surface of the right hind paw, 30 min after the treatment. Animals were then observed individually for 15 min following glutamate injection. The amount of time spent licking and biting the injected paw was timed with a chronometer and considered as an indication of nociception.

### 4.6. Analysis of the Possible Mechanism of Action of Cardamonin

#### 4.6.1. Involvement of l-arginine/Nitric Oxide Pathway

To assess the possible participation of the l-arginine/nitric oxide pathway in the antinociceptive effect of cardamonin in acetic acid test, animals were pre-treated with l-arginine (100 mg/kg; i.p.; a nitric oxide precursor), 15 min before the administration of cardamonin (1 mg/kg; i.p.), N^ω^-nitro-l-arginine (l-NOARG; 20 mg/kg; i.p.; a nitric oxide synthase inhibitor) or vehicle (10 mL/kg; i.p.) as described previously [64,65]. Nociceptive responses against acetic acid were then recorded for 30 min after the administration of cardamonin, l-NOARG or vehicle, began 5 min after the acetic acid injection. The numbers of abdominal writhing were considered as indication of pain behaviour. Another group of animals were pre-treated with vehicle (10 mL/kg; i.p.), and after 15 min, they received cardamonin (1 mg/kg; i.p.), l-NOARG (20 mg/kg; i.p.) or vehicle, 30 min before acetic acid injection.

#### 4.6.2. Involvement of Cyclic Guanosine Monophosphate (cGMP)

To elucidate the possible contribution of cGMP in the antinociceptive effect of cardamonin in the acetic acid test, animals were pre-treated with ODQ (2 mg/kg; i.p.; a selective inhibitor of soluble guanylyl cyclise), 15 min before the administration of cardamonin (1 mg/kg; i.p.) or vehicle (10 mL/kg; i.p.) as described previously [66,67], with slight modifications. Nociceptive responses against acetic acid were then recorded for 30 min after the administration of cardamonin or vehicle, beginning 5 min after the acetic acid injection. The numbers of abdominal writhing were counted as indication of pain behaviour. Another group of animals were pre-treated with vehicle (10 mL/kg; i.p.), and after 15 min, they received cardamonin (1 mg/kg; i.p.) or vehicle, 30 min before acetic acid injection.

#### 4.6.3. Involvement of ATP-Sensitive K^+^ Channel

In order to investigate the possible participation of K^+^ channel in the antinociceptive effect of cardamonin in acetic acid test, animals were pre-treated with glibenclamide (10 mg/kg; i.p.; an ATP-sensitive K^+^ channel inhibitor), 15 min prior to the injection of either cardamonin (1 mg/kg; i.p.) or vehicle (10 mL/kg; i.p.), as described previously [60,64], with slight modifications. The animals were then challenged with acetic acid (i.p.) 30 min after the treatment. The animals were immediately placed into a Perspex chamber after injection of acetic acid and the numbers of abdominal writhing were recorded for 30 min, began 5 min after the acetic acid injection. Another group of animals were pre-treated with vehicle (10 mL/kg; i.p.), and after 15 min, they received cardamonin or vehicle, 30 min before acetic acid injection.

### 4.7. Data Analysis

The data collected were expressed as mean ± S.E.M. for six animals per group and analysed using one-way ANOVA followed by Tukey’s multiple comparison test. The differences between means were considered as statistically significant at *p* < 0.05. The percentage of inhibition was calculated by comparing the results of treatment group with control group.

## 5. Conclusions

Findings in the present study suggested that systemic administration of cardamonin, in doses that does not produce any apparent toxicity and motor impairment in animals, exerted antinociceptive effects through the inhibition of PKC activation, modulation of NMDA and non-NMDA glutamate receptors, interruption of NMDA activation evoked nitric oxide/cGMP/glutamate release cascade, and the modulation of nitric oxide/cGMP-mediated activation of ATP-sensitive potassium channels. The precise mechanism underlying remains to be investigated through molecular analysis.

## References

[1] Rao C.B., Rao T.N., Suryaprakasam S. (1976). Cardamonin and alpinetin from the seeds of Amomum subulatum. Planta Med..

[2] Ahmad S., Israf D.A., Lajis N.H., Shaari K., Mohamed H., Wahab A.A., Ariffin K.T., Hoo W.Y., Aziz N.A., Kadir A.A. (2006). Cardamonin, inhibits pro-inflammatory mediators in activated RAW 264.7 cells and whole blood. Eur. J. Pharmacol..

[3] Chow Y.-L., Lee K.-H., Vidyadaran S., Lajis N.H., Akhtar M.N., Israf D.A., Syahida A. (2012). Cardamonin from Alpinia rafflesiana inhibits inflammatory responses in IFN-γ/LPS-stimulated BV2 microglia via NF-ҡB signalling pathway. Int. Immunopharmacol..

[4] Wang Z.-T., Lau C.-W., Chan F.L., Yao X., Chen Z.-Y., He Z.-D., Huang Y. (2001). Vasorelaxant effects of cardamonin and alpinetin from Alpinia henryi K. Schum. J. Cardiovasc. Pharmacol..

[5] Pascoal A.C.R.F., Ehrenfried C.A., Lopez B.G.-C., de Araujo T.M., Pascoal V., Gilioli R., Anhê G.F., Ruiz A.L.T.G., Carvalho J.E.D., Stefanello M.L.A. (2014). Antiproliferative activity and induction of apoptosis in PC-3 cells by the chalcone cardamonin from Campomanesia adamantium (Myrtaceae) in a bioactivity-guided study. Molecules.

[6] Qin Y., Sun C.-Y., Lu F.-R., Shu X.-R., Yang D., Chen L., She X.-M., Gregg N.M., Guo T., Hu Y. (2012). Cardamonin exerts potent activity against multiple myeloma through blockade of NF-KB pathway in vitro. Leuk. Res..

[7] Tang Y., Fang Q., Shi D., Niu P., Chen Y., Deng J. (2012). mTOR inhibition of cardamonin on antiproliferation of A549 cells is involved in a FKBP12 independent fashion. Life Sci..

[8] Wei Z., Yang J., Xia Y.e., Huang W.h., Wang Z.a., Dai Y. (2012). Cardamonin protects septic mice from acute lung injury by preventing endothelial barrier dysfunction. J. Biochem. Mol. Toxicol..

[9] Trakoontivakorn G., Nakahara K., Shinmoto H., Takenaka M., Onishi-Kameyama M., Ono H., Yoshida M., Nagata T., Tsushida T. (2001). Structural analysis of a novel antimutagenic compound, 4-hydroxypanduratin A, and the antimutagenic activity of flavonoids in a Thai spice, fingerroot (Boesenbergia pandurata Schult.) against mutagenic heterocyclic amines. J. Agric. Food Chem..

[10] Liao Q., Shi D.-H., Zheng W., Xu X.-J., Yu Y.-H. (2010). Antiproliferation of cardamonin is involved in mTOR on aortic smooth muscle cells in high fructose-induced insulin resistance rats. Eur. J. Pharmacol..

[11] Cho M., Ryu M., Jeong Y., Chung Y.-H., Kim D.-E., Cho H.-S., Kang S., Han J.-S., Chang M.-Y., Lee C.-K. (2009). Cardamonin suppresses melanogenesis by inhibition of Wnt/β-catenin signaling. Biochem. Biophys. Res. Commun..

[12] Park M.K., Choi J.K., Kim H.J., Nakahata N., Lim K.M., Kim S.Y., Lee C.H. (2014). Novel inhibitory effects of cardamonin on thromboxane A2-induced scratching response: Blocking of Gh/transglutaminase-2 binding to thromboxane A2 receptor. Pharmacol. Biochem. Behav..

[13] El-Naga R.N. (2014). Pre-treatment with cardamonin protects against cisplatin-induced nephrotoxicity in rats: Impact on NOX-1, inflammation and apoptosis. Toxicol. Appl. Pharmacol..

[14] Zhang T., Yamamoto N., Yamashita Y., Ashida H. (2014). The chalcones cardamonin and flavokawain B inhibit the differentiation of preadipocytes to adipocytes by activating ERK. Arch. Biochem. Biophys..

[15] Mohammed I.A., Akhtar M.N., Biau F.J., Tor Y.S., Zareen S., Binti Shahabudin S., Binti Abd Hamid H., Ul Haq Z., Khalil R., Khalaf R.M. (2019). Isolation of Cardamonin and Pinostrobin Chalcone from the Rhizomes of Boesenbergia rotunda (L.) Mansf. and their Cytotoxic Effects on H-29 and MDA-MB-231 Cancer Cell Lines. Nat. Prod. J..

[16] Niu P., Shi D., Zhang S., Zhu Y., Zhou J. (2018). Cardamonin enhances the anti-proliferative effect of cisplatin on ovarian cancer. Oncol. Lett..

[17] Hou S., Yuan Q., Yu N., Liu B., Huang G., Yuan X. (2019). Cardamonin attenuates chronic inflammation and tumorigenesis in colon. Cell Cycle.

[18] Park M.K., Jo S.H., Lee H.J., Kang J.H., Kim Y.R., Kim H.J., Lee E.J., Koh J.Y., Ahn K.O., Jung K.C. (2013). Novel suppressive effects of cardamonin on the activity and expression of transglutaminase-2 lead to blocking the migration and invasion of cancer cells. Life Sci..

[19] James S., Aparna J.S., Paul A.M., Lankadasari M.B., Mohammed S., Binu V.S., Santhoshkumar T.R., Reshmi G., Harikumar K.B. (2017). Cardamonin inhibits colonic neoplasia through modulation of MicroRNA expression. Sci. Rep..

[20] Jin J., Qiu S., Wang P., Liang X., Huang F., Wu H., Zhang B., Zhang W., Tian X., Xu R. (2019). Cardamonin inhibits breast cancer growth by repressing HIF-1α-dependent metabolic reprogramming. J. Exp. Clin. Cancer Res..

[21] Hou G., Yuan X., Li Y., Hou G., Liu X. (2020). Cardamonin, a natural chalcone, reduces 5-fluorouracil resistance of gastric cancer cells through targeting Wnt/β-catenin signal pathway. Investig. New Drugs.

[22] Wang Z., Tang X., Wu X., Yang M., Wang W., Wang L., Tang D., Wang D. (2019). Cardamonin exerts anti-gastric cancer activity via inhibiting LncRNA-PVT1-STAT3 axis. Biosci. Rep..

[23] Voon F.-L., Sulaiman M.R., Akhtar M.N., Idris M.F., Akira A., Perimal E.K., Israf D.A., Ming-Tatt L. (2017). Cardamonin (2′-4′-dihydroxy-6′-methoxychalcone) isolated from Boesenbergia rotunda (L.) Mansf. inhibits CFA-induced rheumatoid arthritis in rats. Eur. J. Pharmacol..

[24] Sambasevam Y., Farouk A.A.O., Mohamad T.A.S.T., Sulaiman M.R., Bharatham B.H., Perimal E.K. (2017). Cardamonin attenuates hyperalgesia and allodynia in a mouse model of chronic constriction injury-induced neuropathic pain: Possible involvement of the opioid system. Eur. J. Pharmacol..

[25] Benchabane S., Belguendouz H., Behairi N., Arroul-Lammali A., Boudjelida A., Youinou P., Touil-boukoffa C. (2018). Cardamonin inhibits pro-inflammatory cytokine production and suppresses NO pathway in PBMCs from patients with primary Sjogren’s syndrome. Immunopharmacol. Immunotoxicol..

[26] Israf D.A., Khaizurin T.A., Syahida A., Lajis N.H., Khozirah S. (2007). Cardamonin inhibits COX and iNOS expression via inhibition of p65NF-ҡB nuclear translocation and ҡ-B phosphorylation in RAW 264.7 macrophage cells. Mol. Immunol..

[27] Takahashi A., Yamamoto N., Murakami A. (2011). Cardamonin suppresses nitric oxide production via blocking the IFN-γ/STAT pathway in endotoxin-challenged peritoneal macrophages of ICR mice. Life Sci..

[28] Park M.K., Lee H.J., Choi J.K., Kim H.J., Kang J.H., Lee E.J., Kim Y.R., Kang J.H., Yoo J.K., Cho H.Y. (2014). Novel anti-nociceptive effects of cardamonin via blocking expression of cyclooxygenase-2 and transglutaminase-2. Pharmacol. Biochem. Behav..

[29] Ping C.P., Mohamad T., Shah T.A., Akhtar M.N., Perimal E.K., Akira A., Ali I., Ahmad D., Sulaiman M.R. (2018). Antinociceptive effects of cardamonin in mice: Possible involvement of TRPV1, glutamate, and opioid receptors. Molecules.

[30] Beirith A., Santos A.R.S., Calixto J.B. (2002). Mechanisms underlying the nociception and paw oedema caused by injection of glutamate into the mouse paw. Brain Res..

[31] Ferreira J., Santos A.R.S., Calixto J.B. (1999). The role of systemic, spinal and supraspinal l-arginine-nitric oxide-cGMP pathway in thermal hyperalgesia caused by intrathecal injection of glutamate in mice. Neuropharmacology.

[32] Wang S., Zhai C., Zhang Y., Yu Y., Zhang Y., Ma L., Li S., Qiao Y. (2016). Cardamonin, a novel antagonist of hTRPA1 cation channel, reveals therapeutic mechanism of pathological pain. Molecules.

[33] Zulazmi N.A., Gopalsamy B., Farouk A.A.O., Sulaiman M.R., Bharatham B.H., Perimal E.K. (2015). Antiallodynic and antihyperalgesic effects of zerumbone on a mouse model of chronic constriction injury-induced neuropathic pain. Fitoterapia.

[34] Moriyama T., Higashi T., Togashi K., Iida T., Segi E., Sugimoto Y., Tominaga T., Narumiya S., Tominaga M. (2005). Sensitization of TRPV1 by EP_1_ and IP reveals peripheral nociceptive mechanism of prostaglandins. Mol. Pain.

[35] Ferreira J., Triches K.M., Medeiros R., Calixto J.B. (2005). Mechanisms involved in the nociception produced by peripheral protein kinase c activation in mice. Pain.

[36] Velazquez K.T., Mohammad H., Sweitzer S.M. (2007). Protein kinase C in pain: Involvement of multiple isoforms. Pharmacol. Res..

[37] Vellani V., Mapplebeck S., Moriondo A., Davis J.B., McNaughton P.A. (2001). Protein kinase C activation potentiates gating of the vanilloid receptor VR1 by capsaicin, protons, heat and anandamide. J. Physiol..

[38] Sikand P., Premkumar L.S. (2007). Potentiation of glutamatergic synaptic transmission by protein kinase C-mediated sensitization of TRPV1 at the first sensory synapse. J. Physiol..

[39] Wang Y., Wu J., Wu Z., Lin Q., Yue Y., Fang L. (2010). Regulation of AMPA receptors in spinal nociception. Mol. Pain.

[40] Alexander S.P.H., Malenka R. (2009). Glutamate. Intercellular Communication in the Nervous System.

[41] Petrenko A.B., Yamakura T., Baba H., Shimoji K. (2003). The role of N-methyl-D-aspartate (NMDA) receptors in pain: A review. Anesth. Analg..

[42] Zhou S., Bonasera L., Carlton S.M. (1996). Peripheral administration of NMDA, AMPA or KA results in pain behaviors in rats. Neuroreport.

[43] Lutfy K., Weber E. (1998). Tolerance develops to the antinociceptive and motor impairing effects of ACEA-1416, a NMDA receptor antagonist, in the formalin and rotarod tests in mice. Pharmacol. Res..

[44] Miclescu A., Gordh T. (2009). Nitric oxide and pain:’something old, something new’. Acta Anaesthesiol. Scand..

[45] Esplugues J.V. (2002). NO as a signalling molecule in the nervous system. Br. J. Pharmacol..

[46] Duarte I.D.G., Ferreira S.H. (2000). l-NAME causes antinociception by stimulation of the arginine-NO-cGMP pathway. Mediat. Inflamm..

[47] Sakurada C., Sugiyama A., Nakayama M., Yonezawa A., Sakurada S., Tan-No K., Kisara K., Sakurada T. (2001). Antinociceptive effect of spinally injected l-NAME on the acute nociceptive response induced by low concentrations of formalin. Neurochem. Int..

[48] Moore P.K., Oluyomi A.O., Babbedge R.C., Wallace P., Hart S.L. (1991). l-NG-nitro arginine methyl ester exhibits antinociceptive activity in the mouse. Br. J. Pharmacol..

[49] Kawamata T., Omote K. (1999). Activation of spinal N-methyl-D-aspartate receptors stimulates a nitric oxide/cyclic guanosine 3, 5-monophosphate/glutamate release cascade in nociceptive signaling. Anesthesiology.

[50] Du X., Wang C., Zhang H. (2011). Activation of ATP-sensitive potassium channels antagonize nociceptive behavior and hyperexcitability of DRG neurons from rats. Mol. Pain.

[51] Du X., Gamper N. (2013). Potassium channels in peripheral pain pathways: Expression, function and therapeutic potential. Curr. Neuropharmacol..

[52] Ocana M., Cendan C.M., Cobos E.J., Entrena J.M., Baeyens J.M. (2004). Potassium channels and pain: Present realities and future opportunities. Eur. J. Pharmacol..

[53] Galeotti N., Ghelardini C., Bartolini A. (2001). Involvement of potassium channels in amitriptyline and clomipramine analgesia. Neuropharmacology.

[54] Hajhashemi V., Amin B. (2011). Effect of glibenclamide on antinociceptive effects of antidepressants of different classes. Clinics.

[55] Soares A.C., Leite R., Tatsuo M.A.K.F., Duarte I.D.G. (2000). Activation of ATP-sensitive K^+^ channels: Mechanism of peripheral antinociceptive action of the nitric oxide donor, sodium nitroprusside. Eur. J. Pharmacol..

[56] Vale M.L., Rolim D.E., Cavalcante I.F., Ribeiro R.A., Souza M. (2007). Role of NO/cGMP/KATP pathway in antinociceptive effect of sildenafil in zymosan writhing response in mice. Inflamm. Res..

[57] Staurengo-Ferrari L., Zarpelon A.C., Longhi-Balbinot D.T., Marchesi M., Cunha T.M., Alves-Filho J.C., Cunha F.Q., Ferreira S.H., Casagrande R., Miranda K.M. (2014). Nitroxyl inhibits overt pain-like behavior in mice: Role of cGMP/PKG/ATP-sensitive potassium channel signaling pathway. Pharmacol. Rep..

[58] Kawano T., Zoga V., Kimura M., Liang M.-Y., Wu H.-E., Gemes G., McCallum J.B., Kwok W.-M., Hogan Q.H., Sarantopoulos C.D. (2009). Nitric oxide activates ATP-sensitive potassium channels in mammalian sensory neurons: Action by direct S-nitrosylation. Mol. Pain.

[59] Sulaiman M.R., Somchit M.N., Israf D.A., Ahmad Z., Moin S. (2004). Antinociceptive effect of Melastoma malabathricum ethanolic extract in mice. Fitoterapia.

[60] Khalid M.H., Akhtar M.N., Mohamad A.S., Perimal E.K., Akira A., Israf D.A., Lajis N., Sulaiman M.R. (2011). Antinociceptive effect of the essential oil of Zingiber zerumbet in mice: Possible mechanisms. J. Ethnopharmacol..

[61] Meotti F.C., Luiz A.P., Pizzolatti M.G., Kassuya C.A.L., Calixto J.B., Santos A.R.S. (2006). Analysis of the antinociceptive effect of the flavonoid myricitrin: Evidence for a role of the l-arginine-nitric oxide and protein kinase C pathways. J. Pharmacol. Exp. Ther..

[62] Schmidt A.P., Tort A.B., Silveira P.P., Bohmer A.E., Hansel G., Knorr L., Schallenberger C., Dalmaz C., Elisabetsky E., Crestana R.H. (2009). The NMDA antagonist MK-801 induces hyperalgesia and increases CSF excitatory amino acids in rats: Reversal by guanosine. Pharmacol. Biochem. Behav..

[63] Yoon M.H., Bae H.B., Choi J.I. (2005). Antinociceptive interactions between intrathecal gabapentin and MK801 or NBQX in rat formalin test. J. Korean Med. Sci..

[64] Perimal E.K., Akhtar M.N., Mohamad A.S., Khalid M.H., Ming O.H., Khalid S., Tatt L.M., Kamaldin M.N., Zakaria Z.A., Israf D.A. (2011). Zerumbone-induced antinociception: Involvement of the l-arginine-nitric oxide-cGMP-PKC-K+ ATP channel pathways. Basic Clin. Pharmacol. Toxicol..

[65] Santos C.A., Santos D.S., Santana D.G., Thomazzi S.M. (2013). Evaluation of mechanisms involved in the antinociception of the ethanol extract from the inner bark of Caesalpinia pyramidalis in mice. J. Ethnopharmacol..

[66] Mohamad A.S., Akhtar M.N., Khalivulla S.I., Perimal E.K., Khalid M.H., Ong H.M., Zareen S., Akira A., Israf D.A., Lajis N. (2011). Possible Participation of Nitric Oxide/Cyclic Guanosine Monophosphate/Protein Kinase C/ATP-Sensitive K^+^ Channels Pathway in the Systemic Antinociception of Flavokawin B. Basic Clin. Pharmacol. Toxicol..

[67] Rangel R.A.S., Marinho B.G., Fernandes P.D., Moura R.S., Lessa M.A. (2014). Pharmacological mechanisms involved in the antinociceptive effects of dexmedetomidine in mice. Fundam. Clin. Pharmacol..

